# System Interdependency Modeling in the Design of Prognostic and Health Management Systems in Smart Manufacturing

**Published:** 2015

**Authors:** M.L. Malinowski, P.A. Beling, Y.Y. Haimes, A. LaViers, J.A. Marvel, B.A. Weiss

**Affiliations:** 1,2,3,4 University of Virginia, Charlottesville, Virginia, 22904, USA; 5,6 National Institute of Standards and Technology, Gaithersburg, Maryland, 20899, USA

## Abstract

The fields of risk analysis and prognostics and health management (PHM) have developed in a largely independent fashion. However, both fields share a common core goal. They aspire to manage future adverse consequences associated with prospective dysfunctions of the systems under consideration due to internal or external forces. This paper describes how two prominent risk analysis theories and methodologies – Hierarchical Holographic Modeling (HHM) and Risk Filtering, Ranking, and Management (RFRM) – can be adapted to support the design of PHM systems in the context of smart manufacturing processes. Specifically, the proposed methodologies will be used to identify targets – components, subsystems, or systems – that would most benefit from a PHM system in regards to achieving the following objectives: minimizing cost, minimizing production/maintenance time, maximizing system remaining usable life (RUL), maximizing product quality, and maximizing product output.

HHM is a comprehensive modeling theory and methodology that is grounded on the premise that no system can be modeled effectively from a single perspective. It can also be used as an inductive method for scenario structuring to identify emergent forced changes (EFCs) in a system. EFCs connote trends in external or internal sources of risk to a system that may adversely affect specific states of the system. An important aspect of proactive risk management includes bolstering the resilience of the system for specific EFCs by appropriately controlling the states. Risk scenarios for specific EFCs can be the basis for the design of prognostic and diagnostic systems that provide real-time predictions and recognition of scenario changes. The HHM methodology includes visual modeling techniques that can enhance stakeholders’ understanding of shared states, resources, objectives and constraints among the interdependent and interconnected subsystems of smart manufacturing systems. In risk analysis, HHM is often paired with Risk Filtering, Ranking, and Management (RFRM). The RFRM process provides the users, (e.g., technology developers, original equipment manufacturers (OEMs), technology integrators, manufacturers), with the most critical risks to the objectives, which can be used to identify the most critical components and subsystems that would most benefit from a PHM system.

A case study is presented in which HHM and RFRM are adapted for PHM in the context of an active manufacturing facility located in the United States. The methodologies help to identify the critical risks to the manufacturing process, and the major components and subsystems that would most benefit from a developed PHM system.

## 1. Introduction

Smart Manufacturing Systems require advanced technologies that facilitate widespread information flow within the system’s components and subsystems. This information can include the health, performance, and risk of the system in failing to meet an objective (Jung, Morris, Lyons, Leong, & Cho, 2015). The engineering focus of Prognostics and Health Management (PHM) is coupled with smart manufacturing. The term “prognostics” refers to the prediction of the future status, health, or performance of components and systems. A commonly used metric within engineering prognostics is the remaining usable life (RUL) of a machine or system (National Institute of Standards and Technology, 2014). The term “health management” on the other hand refers to the process of making maintenance and logistics decisions from the prognostics information, available resources, and operational demand (Barajas & Srinivasa, 2008). The focus of health management is to minimize operational loss and to maximize the objectives established by the facility (Lee, Wu, Zhao, Ghaffari, Liao, & Siegel, 2014).

The use of PHM models to improve manufacturing performance has been demonstrated in numerous case studies within automotive (Holland, Barajas, Salman, & Zhang, 2010), aerospace (Batzel & Swanson, 2009), machine tool (Biehl, Staufenbiel, Recknagel, Denkena, & Bertram, 2012), and power generation (Hofmeister, Wagoner, & Goodman, 2013) industries. However, as manufacturing processes increase in size and complexity, it can become exceedingly difficult to determine which components or subsystems can most benefit from a PHM system model. Even data-driven approaches, which rely on historical data and mathematical models, lose accuracy and become less predictive as complexity increases (Bai, Wang, & Hu, 2015).

When available resources for PHM efforts are limited, designers and implementers of PHM systems face a difficult problem in deciding where to deploy these scarce resources to maximize benefit. A smart manufacturing system may involve multiple subsystems or processes that present reasonable targets for the development of PHM systems (Barajas & Srinivasa, 2008). This selection problem is made more difficult because the potential costs and benefits of those potential PHM systems are subject to random and known uncertainty (Feldman, Jazouli, & Sandborn, 2009) (Hou-bo, & Jian-min, 2011).

Numerous systems-based risk analysis methodologies designed to support decision-makers within manufacturing industries have successfully been developed and deployed (Lee, Lv, & Hong, 2013) (Fernández, & Pérez, 2015), including Hierarchical Holographic Modeling (HHM) (Haimes, 2009) and Risk Filtering, Ranking, and Management (RFRM) (Haimes, Kaplan, & Lambert, 2002) (Haimes, 2009). The original purpose of these methods (within the field of risk analysis) was to identify the most critical sources of risks to a system and to provide risk assessment, risk management, and risk communication (Haimes, 2012). With a few modifications, the critical risks identified in the HHM and RFRM processes can be used to identify the most critical components and subsystems that would most benefit from a PHM system or model.

The contribution of this paper is to introduce HHM and RFRM as methodologies to provide scope and direction for the PHM system designer. The proposed methodologies will be used to identify targets – components, subsystems, or systems – that would most benefit from a PHM system in regards to achieving the following objectives: minimizing cost, minimizing production/maintenance time, maximizing system remaining usable life (RUL), maximizing product quality, and maximizing product output. There currently exist multiple methods to determine the major failure modes of a system after an accident or catastrophe (Cocheteux, Voisin, Levrat, & Iung, 2009) (Lee et al., 2014) (Vykydal, Plura, Halfarová, & Klaput, 2015). The proposed methodology allows for a thorough analysis to be conducted even before a failure occurs in a manufacturing environment.

The remainder of the paper is organized as follows. Section 2 summarizes and explores the general HHM methodology. Section 3 explains the additional benefit of applying RFRM to the models developed using HHM. Section 4 discusses PHM-specific modifications to the RFRM method. Section 5 provides a specific case study of the application of HHM and RFRM to a major manufacturing facility. Section 6 concludes the paper.

## 2. Hierarchical Holographic Modeling, Risk Analysis, and PHM

Risk is a combined measure of the probability and severity of adverse effects (Andretta, 2014), which necessitates knowledge and understanding of future probable adverse events and their likely consequences (Haimes, 2009). To answer the basic question in risk analysis: “what can go wrong?” it is imperative that all conceivable and likely risk scenarios be identified. This is a daunting task, but can be accomplished by integrating knowledge and experience from multiple experts across different disciplines. The HHM methodology facilitates this collaboration between experts.

HHM has been successfully utilized in numerous projects and for multiple agencies, including the President’s Commission on Critical Infrastructure Protection (PCCIP), the Federal Bureau of Investigation (FBI), the National Aeronautics and Space Administration (NASA), the Virginia Department of Transportation (VDOT), and the U.S. Army National Ground Intelligence Center (NGIC) (Haimes, 2009) (Lambert, Haimes, Li, Schooff, & Tulsiani, 2001). The PCCIP utilized HHM to determine the major hardware, software, human, and environmental risks to a supervisory control and data acquisition system (Chittester, & Haimes, 2004). The FBI developed an HHM model to identify varying perspectives, motives, and weaknesses between homeland defenders and terrorist networks (Haimes, & Horowitz, 2004). For VDOT, the HHM method identified major interdependencies within Virginia’s transportation infrastructure and outlined critical sectors that were most sensitive to disruptions (Crowther, Dicdican, Leung, Lian, & Williams, 2004). Finally for the Army NGIC, HHM was used prior to a major deployment to identify the critical state variables of the target host country, U.S. forces, and U.S. allies (Dombroski, Haimes, Lambert, Schlussel, & Sulcoski, 2002).

Haimes (2009) defines HHM as a holistic philosophy and methodology aimed at capturing and representing the essence of the inherent diverse characteristics and attributes of a system. These system attributes include, but are not limited to, the multiple aspects, perspectives, facets, views, dimensions, and hierarchies. The mathematical and systems approach to holographic modeling reveals the interconnectedness, and the interdependencies among the system’s objective functions, constraints, decision variables, and inputs/outputs (Haimes, 2009). The term holographic refers to the desire to have a multi-view image of a system (Crowther et al., 2004). For example, the risk to a system due to emergent forced changes (EFCs) can be represented from its multiple perspectives, which are related to time and geography, and include, but are not limited to: (1) economic, (2) health, (3) technical, (4) political, and (5) social perspectives. To capture a holographic outcome, the modeling team that performs the analysis must represent a broad array of experience and knowledge (Haimes, 2009).

The HHM process considers risks at both the macroscopic (management) and microscopic (component) levels. Most organizational and technology-based manufacturing systems are hierarchical in nature (Alvandi, Bienert, Li, & Kara, 2015) (He, Zhang, & Li, 2014), and the deployments of HHM have effectively addressed the risks at these multiple levels (Haimes, Kaplan, & Lambert, 2002). HHM is especially useful in determining the reliability and maintainability of infrastructures that feature a large number of components and subsystems. From a mathematical standpoint, *reliability* refers to the probability that a system is operational in a given time period, while *maintainability* is defined as the probability that a failed system can be restored to an operational state within a specified period of time (Haimes, 2009). Both of these metrics are essential to holistic risk assessment and management.

The HHM methodology produces a multilevel decomposition of a system into its many subsystems and components. This breakdown is essential to revealing the complexity and internal hierarchical nature of large-scale systems (He et al., 2014). Decomposition also allows for trade-off analyses and studies to be performed at the component, subsystem, or total system level. Applying the HHM methodology requires an organized team of experts with varied experience and knowledge bases to develop a holographic view of a system with its multiple levels and hierarchies. Although it is possible for individual experts to create different decompositions, the aggregate will yield the same optimal solution. Each expert will provide their own perspective to enforce the desired multi-view image of the system and reveal unique vulnerabilities (Kaplan, Haimes, & Garrick, 2001). Two major types of risks and uncertainties will ultimately come to light: those resulting from 1) exogenous events such as new legislation or natural disasters, and 2) endogenous events such as hardware, software, organizational, and human failures (Haimes, 2009). While knowledge of both types of events is crucial to understanding the entire system, a PHM system will focus more heavily on potential endogenous events which can take the form of critical EFCs.

At their cores, both PHM and risk analysis share two common goals: (1) to ensure that the systems under consideration perform their intended functions and meet their objectives at acceptable tradeoffs and within an acceptable time frame, and (2) to inform decision-makers so they can better predict and respond to faults and failures (Haimes, 2009) (NIST, 2015). Additionally, both practices utilize systemic risk modeling, assessment, management, and communication to achieve their goals (Ahmad & Kamaruddin, 2012) (Al-Habaibeh & Gindy, 2000). Due to these commonalities, the risk analysis theory and methodology of HHM was utilized in a case study to determine the conceivable sources of risk to a system, and finally to help decide where to apply a PHM model within a smart manufacturing facility.

## 3. Risk Filtering, Ranking, and Management

In total risk management, it is necessary to identify, prioritize, assess, and manage potential risk scenarios to a large-scale system. Stakeholders and decision-makers must consider the likelihoods and consequences of each risk to produce acceptable mitigation options. The Risk Filtering, Ranking, and Management (RFRM) methodology offers eight major phases to guide total risk management in an HHM system (Haimes, 2002). The eight phases are: 1 – Scenario Identification, 2 – Scenario Filtering, 3 – Bi-criteria filtering, 4 – Multi-criteria Evaluation, 5 – Quantitative Ranking, 6 – Risk Management, 7 – Safeguarding Against Missing Critical Items, and 8 – Operational Feedback. Details on these eight phases can be found in (Haimes, 2002).

The guiding force behind RFRM is the identification of head topics, which represent major concepts or perspectives of success, and subtopics, which provide detailed requirements or sources of risk (Haimes, 2009). However, it is often impractical to evaluate hundreds of sources of risk when evaluating a large system. Therefore, the risk scenarios and sources should be filtered based on professional experience, expert knowledge, and statistical data. It is also important to consider a variety of risks such as those related to hardware, software, organizational failure, human error, budget, schedule slip, and performance criteria (Haimes, 2002).

The RFRM methodology has been successfully deployed on numerous systems for multiple agencies, including the NASA, the Federal Aviation Administration (FAA), the VDOT, the National Ground Intelligence Center (NGIC), and the Department of Homeland Security (DHS) (Haimes, 2009). NASA used RFRM to identify the most common risk scenarios facing future space missions (e.g., inadequate oversight teams), and to compare management strategies to mitigate those risks (e.g., restructure existing teams or hire external consultants) (Haimes, 2009). For VDOT, the RFRM method ranked and prioritized the potential shutdowns of various transportation infrastructure assets (e.g., roads, highways, or bridges) according to their impacts on state transportation inoperability and economic loss (Crowther et al., 2004). Finally, the Army NGIC used the RFRM method to identify the risk scenarios that allied forces might encounter in a foreign country that occurred with the highest likelihood probability and produced the most severe results (e.g., loss of life or major asset) (Dombroski et al., 2002).

The risk assessment portion of RFRM can be summed up by four major questions (Haimes, 2002):

1. What can go wrong?
2. What is the likelihood of that happening?
3. What are the consequences?
4. What is the time frame?

The risk management portion on the other hand encompasses three complementary questions (Haimes, 2009):

1. What can be done and what are the available options?
2. What are the associated trade-offs in terms of costs, benefits, and risks?
3. What are the impacts of current decisions on future options?

After all relevant and potential risks have been identified as either head topics or subtopics they must be evaluated by three major criteria: resilience, robustness, and redundancy. *Resilience* refers to the ability of a system to recover after an emergency, and can be evaluated by time and resources needed. *Robustness* is the insensitivity of system performance to external stresses, so the ability to resist potential risks. *Redundancy* refers to the ability of extra components or subsystems to take over the functions of failed components or subsystems (Haimes, 2009).

**Figure 1:**
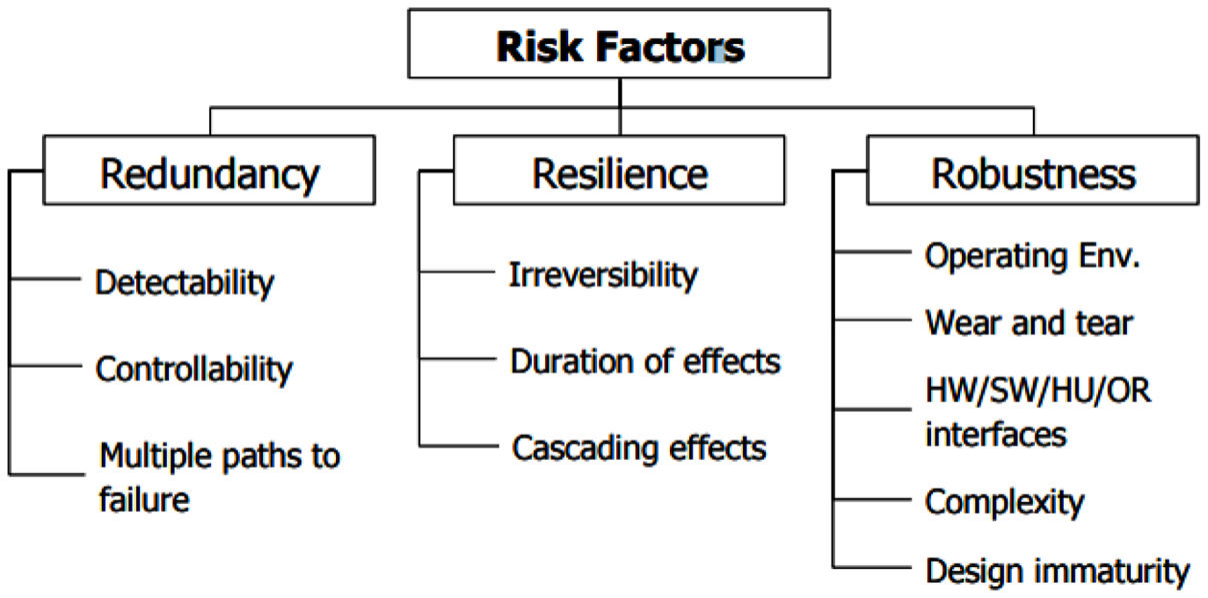
Risk factors with eleven criteria.

The three categories of resilience, robustness, and redundancy are then further broken down into eleven essential criteria for evaluating risk scenarios (refer to Figure 1).

The eleven criteria relating the ability of a risk scenario to defeat the defenses of a system are formally defined as follows (Haimes, 2009):

1. *Undetectability* – the absence of modes by which the initial events of a scenario can be discovered before harm occurs
2. *Uncontrollability* – the absence of control modes that make it possible to take action or make an adjustment to prevent harm
3. *Multiple paths to failure* – multiple and possibly unknown ways for the events of a scenario to harm the system
4. *Irreversibility* – a scenario in which the adverse condition cannot be returned to the initial, operational (pre-event) condition
5. *Duration of effects* – a scenario that would have a long duration of adverse consequences
6. *Cascading effects* – a scenario where the effects of an adverse condition propagate to other systems or subsystems (cannot be contained)
7. *Operating environment* – a scenario that results from external stressors
8. *Wear and tear* – a scenario that results from use, leading to degraded performance
9. *Hardware, software, human, and organizational interfaces* – a scenario in which the adverse outcome is magnified by interfaces among one or more these subsystems
10. *Complexity/emergent behaviors* – a scenario in which there is a potential for system-level behaviors that are not anticipated even with knowledge of components and their interactions
11. *Design immaturity* – a scenario in which the adverse consequences are related to the newness of the system design or other lack of a proven concept

Each identified risk scenario must be rated as “high”, “medium”, “low”, or “not applicable” against each criterion. Scenarios with more “high” ratings must be considered further in the RFRM process. Risk scenarios that score mostly “low” or “not applicable” in the eleven categories can be filtered out unless an emergent change drives it towards a higher level of risk. Alternative rating scales and filtering criteria could also be used with the same goal: reduction of the number of scenarios under consideration.

## 4. PHM-Specific Modifications to Risk Filtering, Ranking, and Management

The RFRM process is essential because it limits the number of risk scenarios for a manufacturing facility to a manageable quantity. However, the process must be modified to identify the risks that are applicable to realistic and practical PHM strategies. Risks that cannot be handled through PHM should still be considered at a higher system level, but will not be useful to the process described in this paper. The modifications to the standard RFRM filtering process are as follows:

M1 Risks that are rated “high” for *undetectability* should be filtered out during RFRM, unless there exists the potential to add a detection method (such as a sensor to a robot).
M2 Risks that are rated “high” for *uncontrollability* should be filtered out during RFRM, unless there exists potential to insert control modes to the process or subsystem.
M3 Risks that are directly related to only the *operating environment* and thus cannot be mitigated on a day-to-day basis should be filtered out during the RFRM.
M4 Risks that can be directly classified as either “human” or “organizational” should be filtered out during RFRM.
M5 Risks that are only rated “high” in the category of design immaturity should be filtered out during RFRM.

The purpose of the M1 modification is to ensure that only risks that can be detected, identified, and diagnosed will remain after the filtering process. This is because PHM systems rely on prognostics, and thus require predictive capabilities of future health, performance, or RUL of subsystems. They must have a means to detect or sense in order to provide effective health management. However, it should be noted that if it is possible to add a detection method or even a reliability model to the risk in question, then it should not be filtered out on the basis of the M1 modification.

The M2 modification seeks to eliminate risks that have no existing control channels. The purpose of a PHM system is to modify decision variables or inputs to a system in order to create a desired outcome. However, even if the optimal modifications to the variables can be identified, if there is no way to implement them, then there is no benefit to the system. It was additionally noted that if it is possible to add control modes, then this filtering criterion can be ignored.

The purpose of the M3 modification is to filter out risks that are *only* related to the operating environment. Specifically, these are the risks pertaining to external factors over which there is no control, such as the weather, plant location, and even legislation or industry standards. These risks should be filtered because they cannot be managed on a day-to-day basis and would require solutions outside the scope of a manufacturing PHM system. It should be noted that this should only serve as a filter if it is the only “high” rated risk category.

Modification M4 removes any risks that are primarily classified as either “human” or “organizational.” The purpose here is to eliminate risks that are primarily related to issues that are difficult to control, such as human error or the organizational structure of a corporation. While managing these risks may prove extremely beneficial to a manufacturing facility, there is little opportunity for a PHM system.

Finally, the M5 modification removes risks that are focused on immature or experimental subsystems, which are usually still undergoing optimization or usability testing. These new systems will naturally inherit additional risk since they have not yet been verified. Therefore, we would not want to allocate resources towards developing a PHM system for a new component until it has become stable within its own design cycle.

## 5. Case Study in the Application of HHM and RFRM to PHM in Smart Manufacturing

The process for identifying the most important sources of risk involves developing a Hierarchical Holographic Model and performing a PHM-oriented Risk Filtering, Ranking, and Management. As a proof of concept for this methodology, consider the following example featuring the packaging process at a major manufacturer located in the United States. Due to the competitive nature of the industry, specific details about the company have been omitted. For the remainder of this paper, the manufacturing facility shall be referred to as Plant A.

### 5.1. Plant A Packing and Bagging Overview

**Figure 2:**
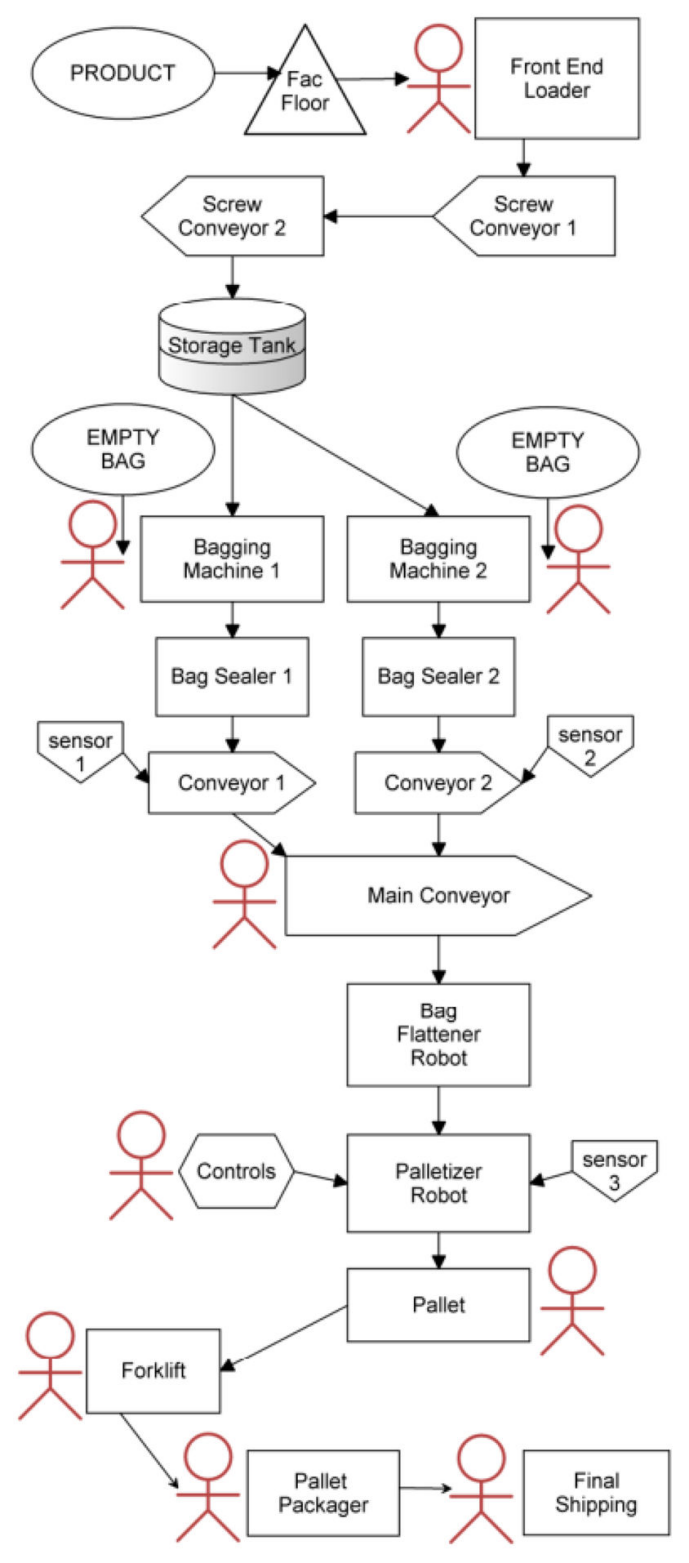
System diagram of Plant A.

One of the major processes at Plant A encompasses the packing, transporting, and bagging of their finished product. Refer to Figure 2 for a detailed system diagram of the entire process with the major components, subsystems, sensors, machines, robots, and humans identified.

Once the product has been processed and fully prepared, it is stored on the floor in a sterilized section of the plant. A small end-loader pushes controlled heaps of the product into a grate in the floor that is outfitted with an automated screw conveyor. This screw moves the product up to a storage tank overhead, which then funnels the product to one of a few bagging stations: two 15.88 kg – 22.68 kg bag stations and one jumbo station for bulk product. After the product enters the funnels, an automated machine fills bags to their correct, preset weight. Bags are administered by human workers, one at each station. The human operators take empty bags, load them onto the filler, and then start the filling process. Finally they remove the full bags and shift the bags over to a conveyor where they are sealed, flattened, and sent down the line.

At this point the bags are in queue for a robotic palletizer. The palletizer receives sealed and inspected bags of product and stacks them onto wooden pallets in regular, repeating patterns that can be selected and adjusted by the operator. A forklift is used to remove the finished pallet where it is wrapped in shrink wrap and placed in a holding area for distribution. A central programmable logic controller with a touch screen interface coordinates the overall unit automation that was supplemented by at least six human workers: one end-loader driver, two baggers, one inspector, and two to shrink wrap finished pallets and insert empty pallets to the palletizer cage. The insertion of empty pallets into the robot workspace is accomplished by a light curtain that would turn off when the pallet was completely loaded (and the robot switched to an empty pallet on its other side) so that the loaded pallet could be removed (via forklift) and a new wooden pallet re-inserted (by a human operator who would return the light curtain to active to let the robot know it could switch back to that side when it finished the pallet on its other side).

Plant engineers have noted the following known health management issues:

- The funnel openings can become clogged with finished product if not regularly cleaned out.
- Sensors fail with regularity. Common causes of failure include occlusion of optical components by dirt and misalignment through collision with bags of product.
- The maneuvering of heavy bags by human workers is a potential source of slower productivity for the facility.
- Adjusting and reprogramming the palletizer is difficult and generally outside the scope of the work done in house. The robot engineer must be on call and able to reprogram the machine in-person.

### 5.2. Application of HHM and RFRM

The main objectives of the manufacturer are to maximize production of their packaged product, and to minimize the risk of a system failure (production shutdown or delay). To help achieve these objectives, Plant A wishes to implement a PHM system into their packing and bagging process. However, they currently have limited monetary resources allocated towards this effort. Thus, Plant A requires a full analysis regarding which of their components/machines/subsystems would most benefit from a PHM system. This necessitates a complete understanding of their current industrial process.

#### 5.2.1. HHM for Plant A

First, multiple Hierarchical Holographic Models (HHMs) are developed covering multiple aspects of the manufacturing plant. The HHM models receive input from many different subject matter experts, stakeholders, and decision makers. For Plant A, an HHM model was originally developed with the perspective of the different *physical components* within the finished product bagging system. The head topics for the model were (1) Machines and Robots, (2) Components, (3) Humans, and (4) Environment. Underneath these major topics, subtopics and possible risk scenarios can be identified. The HHM model for the *physical components* has been displayed in bullet form below.

1. Machines and Robots
  - Front End Loader
  - Screw Conveyor
    - Horizontal
    - Vertical
  - Storage Tank Dispenser
  - Bagging Machine
    - Bag grip
    - Locking mechanism
    - Sensor
    - Product dispenser
  - Bag Sealer
    - Heat sealer
    - Conveyor belt
  - Automated Conveyor
    - Sensor
    - Belt
  - Bag Flattener
  - Palletizer
    - Sensor
    - Arm
    - Claw
    - Controls
  - Forklift
  - Pallet Packager
2. Components
  - Finished Product
  - Bags
  - Pallets
  - Packaging material
3. Humans
  - Front end loader driver
  - Baggers
  - Inspectors
  - Forklift driver
  - Packager
4. Environment
  - Factory Floor
  - Storage Tank
  - Air
  - Moisture
  - Contaminants

A similar HHM model was also developed from multiple experts covering a new perspective: the different *processes* within the finished product bagging system. The practice of creating multiple HHM models helps to provide a holographic view of the entire system and ensure that the major sources of risk are properly captured. It provides a more realistic and complete overall model by recognizing the limitations of modeling a complex system with just a single structure. The head topics for the *processes* model were (1) Storing Product, (2) Transporting Product, (3) Bagging Product, (4) Sealing Bags, (5) Transporting Bags, (6) Flattening Bags, (7) Stacking Bags on a Pallet, and (8) Preparing Final Product for Delivery. The complete HHM model can be seen in bullet form below.

1. Storing Product
  - Environment
    - Factory floor
    - Air
    - Moisture
  - Human interactions
  - Factory contaminant controls
2. Transporting Product
  - Front end loader
    - Scoop product
    - Push product into floor grates
  - Screw conveyors
    - Move product to vertical conveyor
    - Move product to storage tank
3. Bagging Product
  - Human operator
    - Obtain empty bag
    - Fill bag
  - Bagging machine
    - Grip bag
    - Lock bag
    - Sense weight
    - Unlock bag
  - Storage tank
    - Open hatch to drop product
    - Close hatch to secure product
4. Sealing Bags
  - Human operator
    - Place bag
  - Bag sealer
    - Sense bag
    - Grip bag
    - Heat seal bag
    - Transport bag
    - Lay bag flat
5. Transporting Bags
  - Human supervisor
    - Controls
    - Fix unaligned bags
  - Automated conveyor
    - Sense bags
    - Move bags
    - Delay bags
6. Flattening Bags
  - Human supervisor
    - Controls
  - Bag flattener
    - Sense bag
    - Flatten bag
    - Move bag
7. Stacking Bags on a Pallet
  - Forklift
    - Move empty pallet to palletizer
  - Human supervisor
    - Adjust settings for palletizer
    - Start/stop process
    - Fix fallen bags
  - Palletizer robot
    - Sense bag
    - Grip bag
    - Lift bag
    - Position bag
    - Drop/place bag on pallet
8. Preparing Final Product for Delivery
  - Forklift
    - Lift pallet with stacked bags
    - Transport to packager
  - Pallet packager
    - Rotate pallet
    - Dispense shrink wrap
  - Human operator
    - Operate machinery
    - Transport completed pallet to storage area

Multiple HHM perspectives can be explored to further improve the overall system model, such as organizational, technological, or even social. For this particular case study, the *processes* perspective was used to develop risk scenarios for the finished product packing and bagging system.

#### 5.2.2. RFRM for Plant A

Next the Risk Filtering, Ranking, & Management (RFRM) method was applied to the HHM model containing the *processes* within the product packing and bagging system. Each head topic was re-defined as a risk scenario, where the process in question failed to occur. Head topics 2 through 8 were identified as being the most critical to the success of the manufacturing system. The subtopics directly related to the operating environment or human interactions were then filtered out, as per the PHM-specific RFRM modifications. The remaining risk scenarios of interest are identified in Table 1 below.

Next a qualitative severity-scale matrix was applied to the remaining subtopics to filter out the topics that did not meet a predetermined risk threshold. A combination of expert insight from the manufacturers and historic data provided both the evidence for the evaluation and the severity of the impact levels. The results of the matrix are displayed in Table 2 below. The five likelihood/probability columns refer to the probability that an event would normally occur. For example, events in the first column occur with a probability of less than 1%, while events in the second column occur with a probability between 1% and 5%. The descriptions for the matrix scales are displayed in Table 3 and Table 4 below.

According to the RFRM methodology, the topics classified as either “High Risk” or “Extremely High Risk” must be further evaluated, while the other scenarios can be filtered out. In this case, the remaining risk scenarios were:

3.b – Bagging machine failure
6.b – Bag flattener failure
7.c – Palletizer robot failure

These scenarios must be analyzed for their ability to defeat the major defensive properties of a system: redundancy, resilience, and robustness. This can be determined by rating their performance along the eleven criteria RFRM attributes of risk scenarios, displayed in Table 5.

Each category receives a qualitative assessment regarding whether the risk scenario has a low, medium, or high susceptibility to the given criterion. The evaluation for the three remaining risk scenarios within the Plant A example can be seen below (refer to Table 6).

Finally, the PHM-specific RFRM modifications must be checked against the three identified risk scenarios. It can be seen that the palletizer robot failure (7.c) rated high for *undetectability* (1), so according to the PHM modifications it should be removed. However in this case we opt to keep this risk scenario since there are sensors available which can be added to the palletizer robot as detection methods. Additionally the bagging machine failure (3.b) received a high rating for *hardware/software/human/organizational* (9), but only because it was classified as a strictly “human” process. For this reason this risk scenario can be filtered out before further analysis.

#### 5.2.3. Results and Findings from HHM and RFRM

After eliminating risks using the PHM-specific RFRM rules, the palletizer robot received the highest risk assessment both in the qualitative severity-scale matrix (refer to Table 2) and within the eleven attributes of risk (refer to Table 6). Therefore, the HHM and RFRM methodologies have successfully identified an essential location within the Plant A bagging and packaging process. We are confident that the application of a PHM effort at the palletizer robot will provide the biggest impact towards achieving the main objectives: maximizing production and minimizing the risk of a system failure (production shutdown or delay).

Given limited resources, it is recommended that the product manufacturer begin by implementing a PHM strategy at the palletizer robot, and then if available resources remain, proceed with the other top identified sources of risk. The components of the palletizer (arms, claws, sensors, controls, etc.) can even be evaluated for their individual levels of risk to determine which ones are most critical to the palletizer subsystem. Then a variety of PHM methodologies can be implemented for the palletizer to develop an optimal risk management solution for the entire smart manufacturing system-of-systems. This analysis may be crucial in the development of low-level process management by creating awareness of the interconnected system of systems that manufacturing plants rely on to operate efficiently, safely, and in a timely manner. This holistic understanding should trickle down to inform the structure and communications of future robotic control architecture.

## 6. Conclusion

As smart manufacturing facilities increase in size and complexity, it becomes exceedingly challenging to apply Prognostics and Health Management (PHM) models and strategies to the entire system without recognizing and addressing this emergent complexity as a system of systems. This paper has described a systems-based risk-analysis methodology capable of identifying all conceivable sources of risk to smart manufacturing process in support of PHM.

The well-developed practice of risk analysis provides two powerful tools for this methodology: HHM and RFRM. The original purpose of these methods within the risk analysis field was to identify the most critical risks to a system and to provide risk assessment, risk management, and risk communication. However as demonstrated in this paper, with a few modifications the critical risks identified in the HHM and RFRM processes can provide scope and direction for the PHM system designer. Specifically, HHM and RFRM can be utilized to identify the major components, subsystems, or systems that would most benefit from a PHM system while prioritizing the following manufacturing objectives: minimizing cost, minimizing production and maintenance time, maximizing system remaining usable life (RUL), maximizing product quality, and maximizing product output.

## Figures and Tables

**Table 1:** Risk scenarios of interest for RFRM

Risk ID	Risk Description
2.b	Screw conveyor failure
3.b	Bagging machine failure
3.c	Storage tank failure
4.b	Bag sealer failure
5.b	Automated conveyor failure
6.b	Bag flattener failure
7.c	Palletizer robot failure
8.b	Pallet packager failure

**Table 2:** Severity-scale matrix for identified risk scenarios.

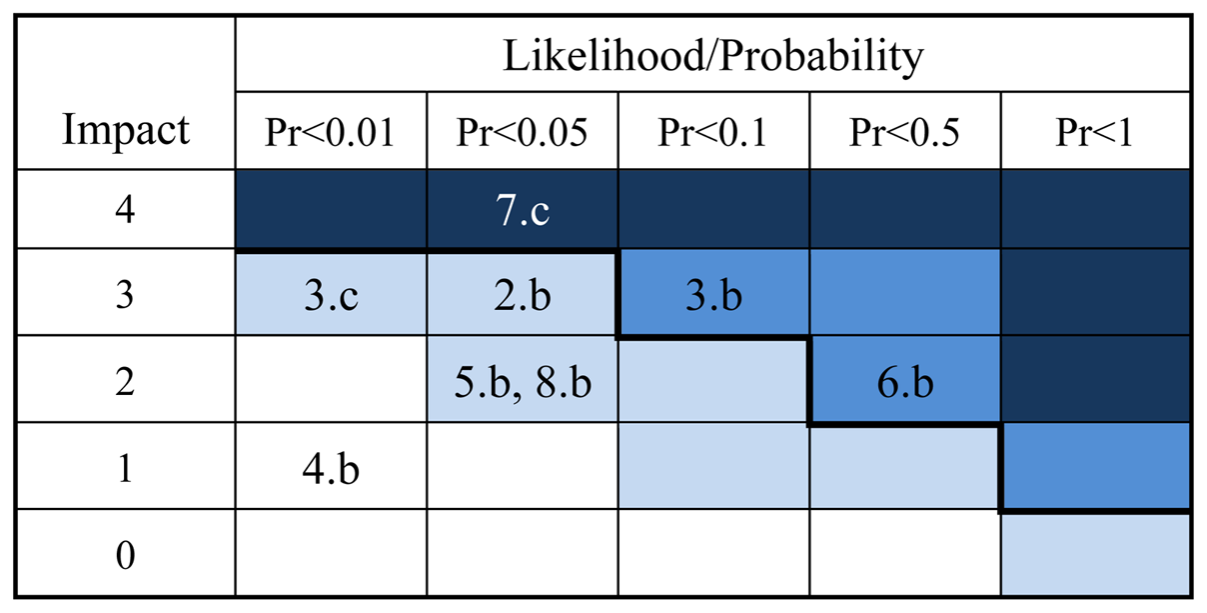

**Table 3:** Risk description for severity matrix

Low Risk	Moderate Risk	High Risk	Extremely High Risk

**Table 4:** Impact description for severity-scale matrix

Impact #	Impact Description
4	Entire Production Shutdown
3	Loss of Product
2	Reduced Production Speed
1	Minor Equipment Degradation
0	Minor or No Effect

**Table 5:** Eleven RFRM attributes of risk scenarios.

#	Criteria
1	Undetectability
2	Uncontrollability
3	Multiple paths to failure
4	Irreversibility
5	Duration of effects
6	Cascading effects
7	Operating environment
8	Wear and tear
9	Hardware/software/human/organizational
10	Complexity and emergent behaviors
11	Design immaturity

**Table 6:** Assessment of risk scenarios using eleven criteria.

Criteria #	3.b Bagging	6.b Flatten	7.c Palletize
1	Medium	Medium	High
2	Low	Low	Low
3	Medium	Low	High
4	Medium	Low	Medium
5	Low	Medium	Medium
6	Medium	Medium	High
7	Medium	Low	Medium
8	Medium	Medium	Medium
9	High (human)	Low	High
10	Low	Low	Medium
11	Low	Low	Low
